# New insights into pathogen-mediated modulation of host RNA splicing

**DOI:** 10.1007/s44154-022-00053-2

**Published:** 2022-08-15

**Authors:** Chuyun Gao, Suomeng Dong

**Affiliations:** Department of Plant Pathology and Key Laboratory of Plant Immunity, Nanjing Agricultural University, Nanjing, 210095 China

**Keywords:** pre-mRNA splicing, plant-microbe interaction, effector, splicing factor

## Abstract

Alternative splicing (AS) regulation of pre-mRNA has been proven to be one of the fundamental layers of plant immune system. How pathogens disrupt plant AS process to suppress plant immunity by secreted effectors remain poorly understood. In the recent study, Gui et al. revealed that a previously identified effector PSR1 of *Phytophthora* interferes with host RNA splicing machinery to modulate small RNA biogenesis, leading to compromised plant immunity. The study provided a novel insight into the importance of AS process during pathogen-host interactions.

## Main text

In eukaryotic cells, the messenger RNA precursor (pre-mRNA) comprises exons for encoding protein sequence, and introns that are in general separated by exons and are considered as non-coding sequence. The spliceosome, a highly dynamic and sophisticated macromolecular machinery, is responsible for the faithful removal of introns from pre-mRNA at the co-transcriptional or post-transcriptional level. The pre-mRNA splicing process may generate different transcript isoforms through alternative splicing (AS). Emerging evidences demonstrated that AS not only increases proteome diversity but also enhances transcriptome complexity and regulation, leading to improved resilience in plant response to environmental changes (Jia et al., 2020). Furthermore, it had been known that many plant key regulators, such as immune receptors, phytochrome receptors and transcription factors, are subjected to AS (Rigo et al., 2019). And, the aberrant AS patterns are frequently associated with development and immune dysfunction (Ren et al., 2021).

Microbes deliver an arsenal of effectors into host cells to manipulate plant immunity by perturbing diverse cellular processes including RNA metabolisms. In 2013, Qiao and colleagues firstly identified two effectors with inhibiting host RNA silencing activity from *Phytophthora sojae*, and revealed a novel virulence mechanism of oomycete pathogens (Qiao et al., 2013). One of these two effectors, *Phytophthora* Suppressor of RNA silencing 1 (PSR1), suppresses plant RNA silencing process by inhibiting small RNAs biogenesis to promote infection. Furthermore, a plant nuclear RNA helicase (PINP1) was identified as a bona fide host target of PSR1 (Qiao et al., 2015). Silencing of *PINP1* leads to developmental defects and susceptibility to *Phytophthora* in Arabidopsis. Interestingly, the localization of the Dicer-like 1 protein complex is impaired in the nucleus of *PINP1*-silenced cells, leading the authors to speculate that PINP1 possibly mediated small RNA metabolism by promoting dicing complex assembly at that time.

In a recent publication, Gui et al. performed an elegant study to test this hypothesis. They generated *P. sojae PSR1* mutants and found that PSR1 is an important virulence factor of *P. sojae* (Gui et al., 2022). PINP1 is the homolog of PRP16, an evolutionarily conserved pre-mRNA splicing factor, and the conserved C-terminus of PINP1 is targeted by the pathogen effector PSR1. Protein structural analysis revealed that human PRP16 is a regulator which functions during remodeling of C-complex in spliceosome to ligate exons after intron splicing (Zhan et al., 2022). Remarkably, the knockdown of *PRP16* induced apoptosis and tumorigenesis in tumor cells (Cona et al., 2022). Gui et al (2022) found that PINP1 possesses not only the binding activity of the long (80-nt) single-strand RNA (ssRNA) and double-strand RNA (dsRNA), but also the dsRNA unwinding activity. Furthermore, the authors found that PINP1 binds to two plant miRNAs, pri-miR172a and pri-miR159b, which involved in growth and stress tolerance (Li et al., 2016; Pan et al., 2016). The RNA binding activity of PINP1 was significantly inhibited by PSR1, thereby impeding sRNA biogenesis. To further explore the underlying mechanism of the PSR1-PINIP1 interaction, authors mined the RNA-Seq data and found that overexpression of PSR1, similar to the silencing of *PINP1*, induced over 8000 differential AS events, among which intron retention (IR) events were the most predominant AS type. They further validated the IR events that are closely relevant to sRNA biogenesis, jasmonic acid (JA) pathway and RNA splicing, and further focused on genes encoding AGO4 and AOC2. Unlike the fully spliced variants, *AGO4* and *AOC2* IR variants induced by PSR1 no longer promote plant resistance. Taken together, these findings suggest that *Phytophthora* PSR1 effector blocks the function of PINP1 and manipulates host RNA splicing to suppress plant immunity.

**Fig. 1:**
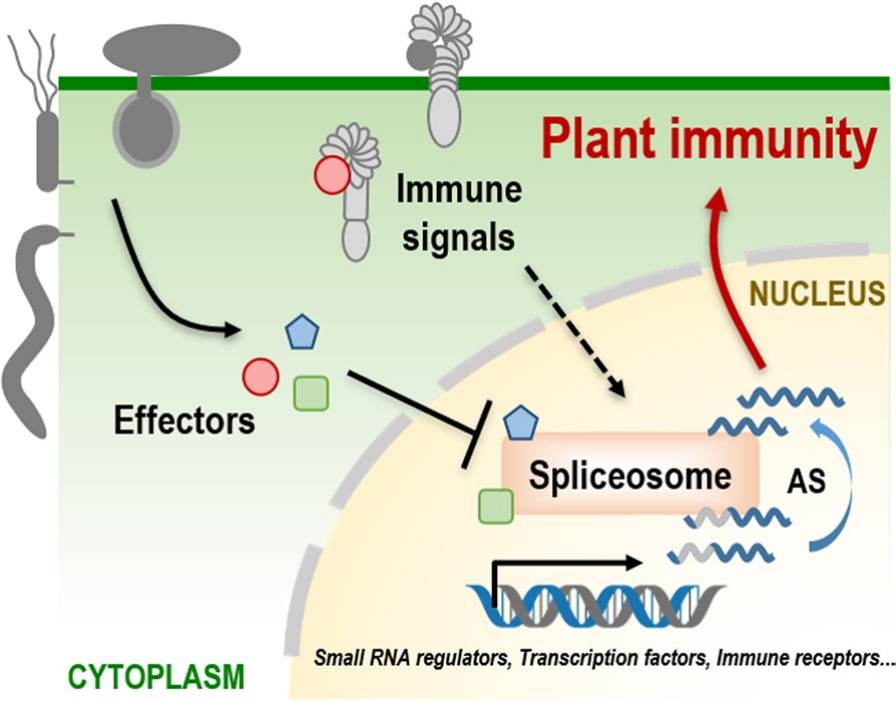
The model of pathogen subverting plant alternative splicing (AS) process to promote infection. When recognizing the pathogen, plants selectively induce alternative splicing on pre-mRNA of immune-related genes, such as small RNA regulators, transcription factors and immune receptors, to activate plant immune system. Meanwhile, pathogens secrete effectors targeting spliceosome or directly as an RNA-binding protein to interfere with plant AS process and suppress plant immunity

To date, plant pathogens from different kingdoms have been demonstrated to modulate host RNA splicing machinery in different ways. The oomycete-secreted effector PsAvr3c physically targets a novel plant splicing regulatory protein SKRP, and alters large-scale host AS dynamics (Huang et al., 2017). The nematode effector 30D08 targets the host spliceosome component SMU2 for parasitism establishment (Verma et al., 2018). Recently, Tang et al. (2022) found that a novel fungal effector Pst_A23 functions like a splicing factor that directly binds to the splicing site of wheat *TaXa21-H* and *TaWRKY53* pre-mRNAs and down regulates functional transcripts to promote host susceptibility (Tang et al., 2022). All these reports, in together with Gui’s latest publication, indicated that AS process is a previously underestimated battlefield during plant and microbe interactions (Figure 1).

It would be exciting to see more studies revealing that pathogens modulate host immunity through interfering RNA splicing homeostasis. A major step towards to this goal is to set up a splicing reporter system and screen for pathogen effectors disrupting plant immunity in a high-throughput manner. Although a few systems in the model plant is available (Huang et al., 2020), other screening methods in other plant species should be developed in the future. Since RNA splicing is one of the fundamental cellular processes, microbe-mediated manipulation of host RNA splicing may interfere with a wild range of signaling and metabolism pathways to enable a favorable nutrient and immune environment for successful infection. Nevertheless, mechanistic investigations of how pathogen manipulate host RNA splicing process may uncover novel models of plant-microbe interactions and provide new strategies in crop breeding for disease and pest resistance.

## Data Availability

Not applicable.

## Abbreviations

pre-mRNA: Messenger RNA precursor
AS: Alternative Splicing
IR: Intron Retention
dsRNA: Double-strand RNA
ssRNA: Single-strand RNA
